# Reduced renal elimination of larger molecules is a strong predictor for mortality

**DOI:** 10.1038/s41598-022-22433-4

**Published:** 2022-10-20

**Authors:** Erik Herou, Anders Grubb, Alain Dardashti, Shahab Nozohoor, Igor Zindovic, Per Ederoth, Henrik Bjursten

**Affiliations:** 1 Department of Cardiothoracic Surgery, Skåne University Hospital, 221 85 Lund, Sweden; 2 Department of Clinical Chemistry, Skåne University Hospital, Lund, Sweden

**Keywords:** Prognostic markers, Risk factors, Glomerulus, Cardiovascular diseases

## Abstract

Renal dysfunction is a major risk factor for premature death and has been studied extensively. A new renal syndrome, shrunken pore syndrome (SPS), confers higher mortality in all studied populations. SPS is a condition in which cystatin C-based estimation of glomerular filtration rate (eGFR_cystatin C_) is ≥ 60% than creatinine-based estimation of glomerular filtration rate (eGFR_creatinine_). We aimed to study the impact of SPS on mortality in a cohort of patients with follow up of up to 10 years. This was a retrospective single centre cohort study. We enrolled 3993 consecutive patients undergoing elective cardiac surgery. Outcome was evaluated using Kaplan Meier analysis and multivariable Cox regression. 1-, 5- and 10-year survival for patients with SPS was 90%, 59% and 45%, and without SPS 98%, 88% and 80% (p < 0.001). SPS was found to be an independent predictor for mortality with an HR of 1.96 (95% CI 1.63–2.36). SPS negatively affected survival regardless of pre-operative renal function. SPS is an independent predictor for mortality after elective cardiac surgery, equal to or greater than risk factors such as diabetes, impaired left ventricular function or renal dysfunction. SPS affected mortality even in patients with normal eGFR.

Clinical registration number: ClinicalTrials.gov, ID NCT04141072.

## Introduction

Renal dysfunction is considered a major risk factor for premature death and has been studied extensively in a multitude of populations^1–3^. Historically, serum creatinine has been the prime surrogate marker for renal function, either as its numerical value or used to estimate the glomerular filtration rate (GFR)^4–6^. In recent years, cystatin C has been used to estimate GFR (eGFR_cystatin C_) either alone or in combination with creatinine and shown to be a superior alternative to creatinine-based estimations of GFR (eGFR_creatinine_) for predicting end-stage renal disease, cardiovascular manifestations and death^7–11^.

Recently, our group reported that a subset of patients with an eGFR_cystatin C_ substantially lower than eGFR_creatinine_ displayed a marked increase in early and mid-term mortality (2–5 years) in elective cardiac surgery patients independent of eGFR^12,13^. We hypothesised that this finding is explained by the kidneys having a reduced clearance for large molecules compared to small molecules, as the molecular weight for creatinine is 113 Da compared to 13,300 Da for cystatin C. Other groups have also reported that this pattern yields a higher mortality in otherwise healthy seniors^14^, a higher incidence of right ventricular failure^15^ and a higher mortality in a mixed patient population with a normal GFR measured by iohexol clearance^16,17^ (Table 1). This pattern where the clearance of cystatin C is reduced compared to that of creatinine has been associated with thickening of the basal membrane in the glomerulus (submitted, available at medRxiv)^18^ and is referred to as Shrunken Pore Syndrome (SPS)^19–21^. We have previously determined that a cut-off of 60% (eGFR_Cystatin C_ ≤ 60% of eGFR_creatinine,_) yields high specificity for premature death^13^.

**Table 1:** Summary of studies on Shrunken Pore Syndrome with clinical outcome data.

Author	Year	N	Population	Results
Grubb et al.	2015	1349	Mixed patient population	SPS was hypothesized
Dardashti et al.	2016	1638	Patients undergoing elective CABG	Higher mortality for patients with SPS
Purde et al.	2016	1467	Healthy seniors	Higher mortality for seniors with SPS
Christensson et al.	2016	143	Patients with heart failure	Patients with SPS more likely to suffer from right heart failure
Herou et al.	2019	4719	Patients undergoing elective CABG and/or sAVR	Higher mortality for patients with SPS
Sällman-Almén et al.	2019	156	Mixed patient population	Higher mortality for patients with SPS
Ljungberg et al.	2019	336	Patients with aortic stenosis	Patients with SPS more likely to require surgery for aortic stenosis
Åkesson et al.	2020	2781	Mixed patient population	Higher mortality for patients with SPS

In this study, we use a large cohort of cardiac surgery patients to investigate mortality of SPS in relation to other risk-factors and laboratory parameters. This population is suited for this analysis as comorbidities are well-documented, pre-operative laboratory parameters are available, and the population has a high prevalence of SPS^13^. Thus, the aim of this study was to investigate the impact SPS has on survival in this cohort and how SPS compares to other risk factors for premature death over a period of 10 years.

## Material and methods

### Trial design

This was a retrospective single centre cohort study with prospective sampling of cystatin C and prospectively collected perioperative data. The study was listed at ClinicalTrials.gov with ID NCT04141072. The regional ethics committee in Lund (LU EPN 2016/53) approved the study and waived informed consent. All methods performed in the article are in accordance with the standard guidelines and regulations^22^. A review of the literature concerning SPS was performed with a search strategy of “SPS” and “Shrunken Pore Syndrome” on pubmed.gov.

### Study population, data collection, blood sampling

**Figure 1:**
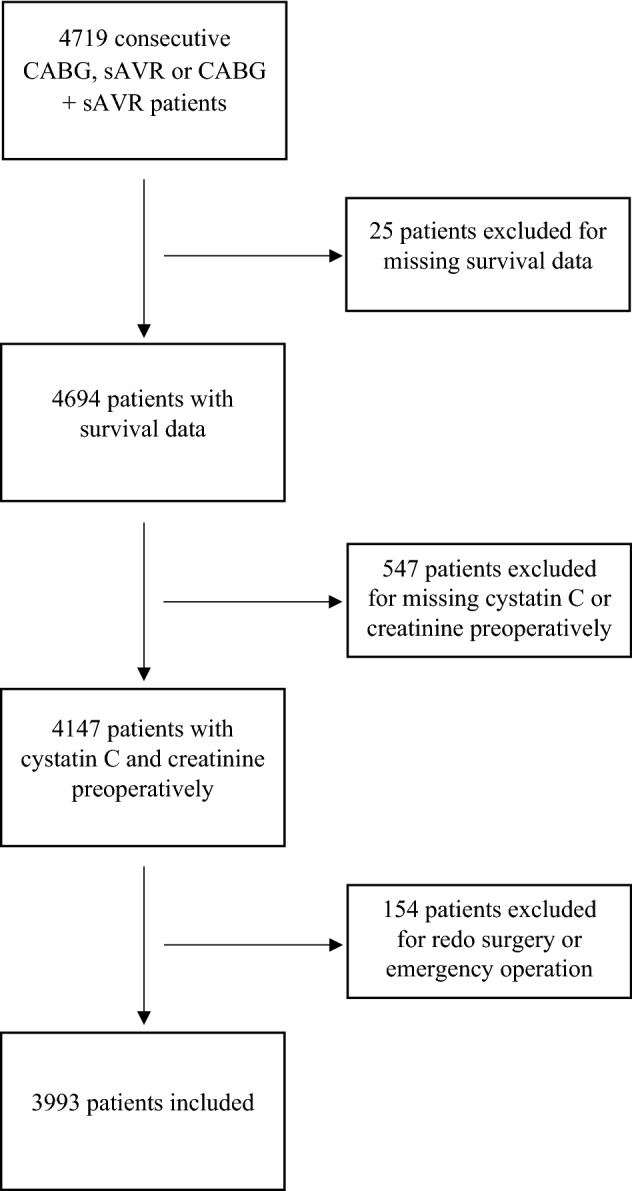
Flow diagram of the study population. *CABG* coronary artery bypass graft, *sAVR* surgical aortic valve replacement.

As previously described^13^ the plasma levels of cystatin C and creatinine were measured simultaneously and preoperatively in 4719 consecutive patients undergoing coronary artery bypass grafting (CABG), surgical aortic valve replacement (SAVR), or CABG + SAVR from 1 January 2010 to 31 December 2015, at the Department of Cardiothoracic Surgery at Skåne University Hospital in Lund, Sweden. See Fig. 1 for a flow diagram of the study population. Perioperative data were obtained from an in-house quality database. Laboratory data were obtained from the hospital clinical chemistry department. Survival data were obtained from the national tax registry in January 2020. Median follow up was 6.5 years (IQR 5.1–8.2) and virtually complete, as the only missing data were from patients who emigrated during the follow-up (emigration of healthy individuals in Sweden in this age category is 0.1%)^23^.

### Definitions and design

The analyses of serum creatinine and serum cystatin C have been described previously^13^. The CKD-EPI equations for estimating GFR based on creatinine (CKD-EPI_creatinine_) or cystatin C (CKD-EPI_cystatin C_) were generally used^4^. The cystatin C-based CAPA- and the creatinine-based LMRev-equations were also used to estimate GFR in a separate analysis which is presented in the Online Appendix^24,25^.

To study the impact of SPS on mortality in different eGFR strata, patients were divided into three groups depending on their pre-operative renal function defined as the mean eGFR from creatinine and cystatin C: normal-mild reduction (eGFR > 60 ml/min/1.73 m^2^), moderate reduction (30–60 ml/min/1.73 m) or severe reduction (eGFR < 30 ml/min1.73 m^2^).

### Statistical analysis

Categorical data were given as proportions, and continuous variables were expressed as the mean ± standard deviation (SD). In skewed distributions, median and interquartile range (IQRs) were reported. Survival rates during follow-up were estimated by Kaplan–Meier curves, and comparisons were performed using log-rank test. Cox multivariable proportional hazards model was used to determine independent predictors for mortality. The variables entered in the Cox proportional hazards regression model were age, sex, body mass index, chronic obstructive pulmonary disease (COPD), creatinine, cystatin C, diabetes, previous cerebrovascular lesion, peripheral vascular disease, anemia (haemoglobin level < 120 g/l), C-reactive protein (CRP), unstable angina pectoris, previous myocardial infarction and ejection fraction ≤ 30%. The variables were chosen as they were readily available and previously shown or hypothesized to influence mortality in cardiac surgery. A backward, stepwise elimination method yielded the risk factors found in Table 2, and these predictors became the final model. This model was used for determining the independent effect of the Shrunken Pore Syndrome by adding SPS to the model. The adjusted Hazard Ratio (HR) and 95% confidence intervals (95% CI) were calculated for Shrunken Pore Syndrome as a predictor. P-values < 0.05 (two-tailed) were considered statistically significant. Statistical analysis was performed using Statistica software version 13.1 (StatSoft Inc., Tulsa, OK), Stata version 14.0 (StataCorp LLC, College Station, TX) and SPSS version 27 (IBM Corp, Armonk, NY).

**Table 2:** Cox univariable and multivariable analysis of risk factors for mortality.

Variable	Univariable analysis		Multivariable analysis	
	P-level	HR (95% CI)	P-level	HR (95% CI)
SPS	< 0.001	3.49 (2.94–4.14)	< 0.001	1.96 (1.63–2.36)
Mean eGFR < 60 ml/min/1.73 m^2^	< 0.001	3.71 (3.25–4.22)	< 0.001	2.82 (2.45–3.24)
Diabetes	< 0.001	1.57 (1.37–1.80)	0.118	1.12 (0.97–1.30)
COPD	< 0.001	1.79 (1.50–2.12)	< 0.001	1.37 (1.14–1.63)
Anaemia	< 0.001	2.86 (2.41–3.40)	< 0.001	1.53 (1.27–1.84)
Peripheral arterial disease	< 0.001	2.38 (2.02–2.80)	< 0.001	1.58 (1.33–1.87)
LVEF < 30%	< 0.001	2.43 (1.99–2.96)	< 0.001	1.55 (1.26–1.90)
Leucocytosis	< 0.001	1.48 (1.29–1.70)	0.026	1.24 (1.08–1.42)
Female sex	< 0.001	1.43 (1.24–1.65)	0.070	1.15 (0.99–1.33)
Age, continuous	< 0.001	1.08 (1.08–1.09)		
CRP	< 0.001	1.01 (1.01–1.01)		
Previous CVI	< 0.001	1.84 (1.53–2.21)		
LVEF < 50%	< 0.001	1.54 (1.34–1.78)		
Mean eGFR, continuous	< 0.001	0.97 (0.96–0.97)		
Mean eGFR < 30 ml/min/1.73 m^2^	< 0.001	4.45 (3.58–5.52)		

*COPD* chronic obstructive pulmonary disease, *CVI* cerebrovascular insult, *eGFR* estimated glomerular filtration rate, *LVEF* left ventricular ejection fraction, *SPS* shrunken pore syndrome.

## Results

**Figure 2:**
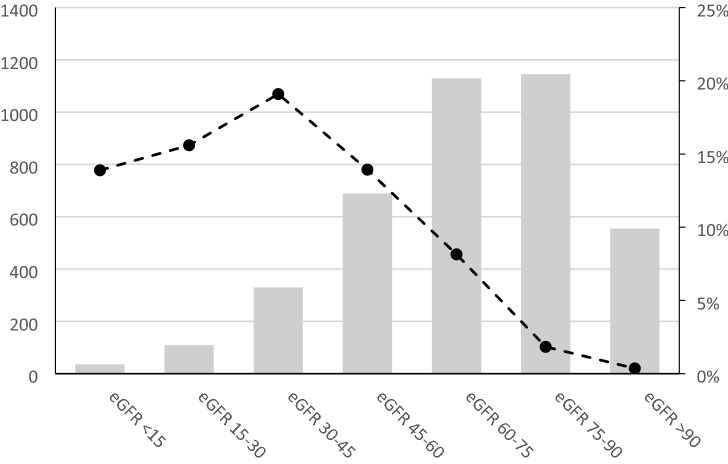
The study population in different mean eGFR strata, all in ml/min/1.73 m^2^, number of patients on the y-axis to the left. The dotted line depicts the distribution of patients with SPS in the different mean eGFR strata in percent on the y-axis to the right. *eGFR* estimated glomerular filtration rate, *SPS* shrunken pore syndrome.

**Figure 3:**
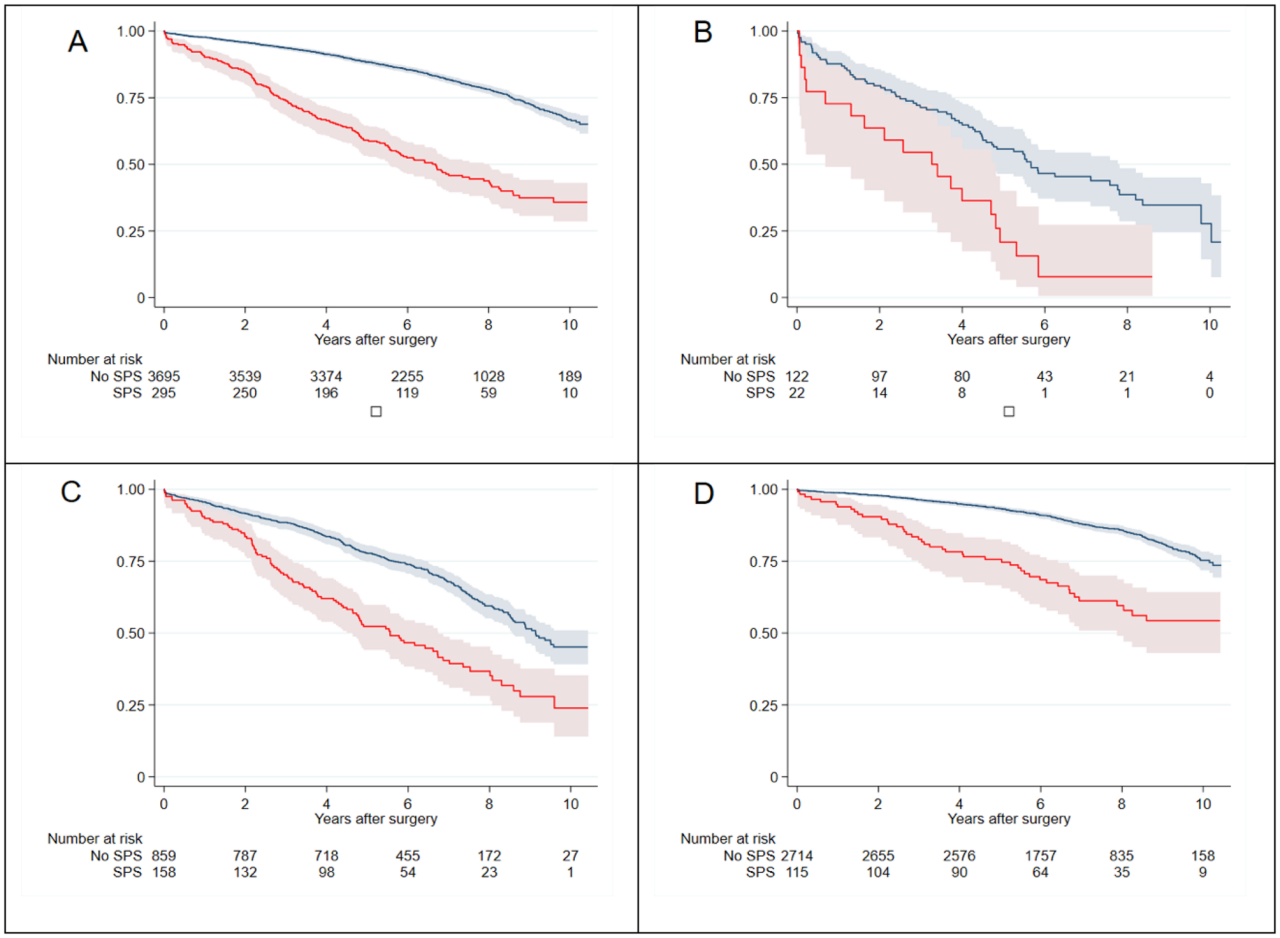
Survival after elective cardiac surgery for all patients, irrespective of eGFR, with Shrunken Pore Syndrome (red solid line) or without SPS (blue solid line) are seen in top left. Patients with eGFR < 30 ml/min/1.73 m^2^ are seen in top right while patients with eGFR 30–60 ml/min/1.73 m^2^ and > 60 ml/min/1.73 m^2^ are seen in bottom left and bottom right, respectively. Faded area represents 95% CI.

Of the 3993 patients in the study, 296 had SPS as defined by eGFR CKD-EPI_cystatin_ ≤ 60% of eGFR CKD-EPI_creatinine_, yielding a prevalence of 7.4%. SPS occurred in patients in different eGFR strata with the highest prevalence of SPS in those with moderately reduced eGFR (30–45 ml/min/1.73 m^2^) (Fig. 2). The 1-, 5- and 10-year survival for the entire cohort was 97%, 86% and 77%; for patients with SPS it was 90%, 59% and 45%; and for patients without SPS it was 98%, 88% and 80% (p < 0.001 in 1-, 5- and 10-year survival) (Fig. 3). Preoperative risk factors, perioperative data and postoperative complications are displayed in the Supplementary material (Supplementary Table S1).

### SPS as risk factor for death

In univariable analysis, SPS yielded a HR for death of 3.49 (95% CI 2.94–4.14) (Table 2). In the multivariable Cox analysis, SPS was found to be an independent predictor for mortality with an HR of 1.96 (95% CI 1.63–2.36). Mean eGFR CKD-EPI < 60 ml/min/1.73 m^2^, COPD, preoperative anaemia, peripheral arterial disease, leucocytosis and left ventricular ejection fraction (LVEF) < 30% were also identified as independent predictors of mortality (Table 2). We performed a test for multicollinearity which showed no collinearity between SPS, Mean eGFR CKD-EPI < 60 ml/min/1.73 m^2^ or any of the other variables included in the Cox model (see Supplementary Table S4).

### SPS in different strata of eGFR, sex and diabetes

The presence of SPS affected survival negatively regardless of strata for pre-operative renal function (mean eGFR < 30, 30–60 and > 60 all in ml/min/1.73 m^2^) as depicted in Fig. 3. Our Cox regression model was employed to test the significance of SPS in the different eGFR strata as well as in each sex and diabetics selectively. The respective Hazard Ratios for SPS with different mean eGFR were 2.74 (95% CI 1.59–4.72), 1.81 (95% CI 1.42–2.31) and 2.16 (95% CI 1.54–3.03), respectively (Table 3).

**Table 3:** Cox multivariable analysis of risk factors for mortality in different eGFR strata; < 30 ml/min/1.73 m^2^, 30–60 ml/min/1.73 m^2^ and > 60 ml/min/1.73 m^2^.

Variables	Mean eGFR < 30 ml/min/1.73 m^2^		Mean eGFR 30–60 ml/min/1.73 m^2^		Mean eGFR > 60 ml/min/1.73 m^2^	
	P-level	HR (95% CI)	P-level	HR (95% CI)	P-level	HR (95% CI)
SPS	< 0.001	2.74 (1.59–4.72)	< 0.001	1.81 (1.42–2.31)	< 0.001	2.16 (1.54–3.03)
Diabetes	0.429	0.84 (0.54–1.30)	0.650	1.05 (0.85–1.30)	0.085	1.21 (0.97–1.52)
COPD	0.793	0.92 (0.50–1.70)	0.033	1.32 (1.02–1.71)	0.004	1.49 (1.14–1.96)
Anaemia	0.696	1.10 (0.69–1.74)	0.006	1.42 (1.10–1.82)	< 0.001	1.97 (1.40–2.77)
Peripheral arterial disease	0.005	1.93 (1.22–3.06)	0.013	1.37 (1.07–1.76)	< 0.001	1.74 (1.31–2.29)
LVEF < 30%	0.008	2.38 (1.25–4.52)	0.009	1.46 (1.10–1.93)	0.014	1.56 (1.09–2.23)
Leucocytosis	0.371	1.22 (0.79–1.89)	0.026	1.26 (1.03–1.55)	0.180	1.16 (0.93–1.43)
Female sex	0.286	1.26 (0.82–1.94)	0.971	1.00 (0.81–1.25)	0.055	1.25 (0.99–1.58)

*COPD* chronic obstructive pulmonary disease, *eGFR* estimated glomerular filtration rate, *LVEF* left ventricular ejection fraction, *SPS* shrunken pore syndrome.

There was no difference in frequency of SPS between sexes: 67 females (7.2%) were afflicted by SPS and 229 males (7.5%). Our main Cox model was employed with males presenting with a HR of 1.95 (1.58–2.43) and females with a HR of 1.94 (1.36–2.76) (Supplementary Table S2 and Supplementary Figs. S1 and S2).

**Figure 4:**
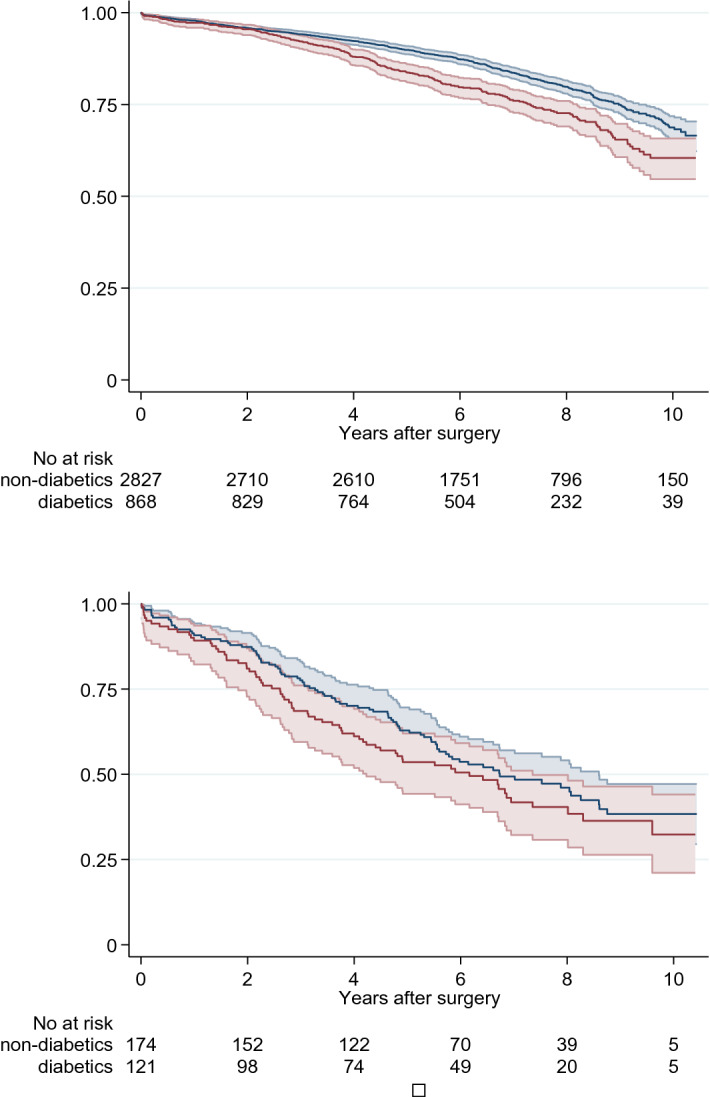
Survival for patients with and without diabetes but without Shrunken Pore Syndrome after elective cardiac surgery are seen in top graph and with Shrunken Pore syndrome are seen in lower graph, diabetic; red solid line, non-diabetic; blue solid line. Faded area represents 95% CI.

We observed a greater proportion of SPS in diabetics, where 121 of all diabetics (12.2%) had SPS while 175 of all non-diabetic patients (5.8%) had SPS (p < 0.001). The difference persisted numerically regardless of strata for pre-operative renal function (see Supplementary material). Our Cox regression model was employed to test the significance of SPS in diabetics and non-diabetics, respectively. The hazard ratios for SPS in diabetics and non-diabetics were 1.76 (95% CI 1.32–2.35) and 2.09 (95% CI 1.65–2.65), respectively (Supplementary Table 3). Further, survival curves for patients with and without diabetes stratified for SPS positive and negative are seen in Fig. 4.

In a separate analysis, we employed the CAPA/LMrev method for estimating GFR as it is calibrated to a Swedish mixed population and therefore better represents the true GFR. In this analysis, the hazard ratio for SPS was virtually the same as presented above (see Supplementary Material).

## Discussion

This study demonstrates that the expected long-term survival rate for patients with SPS is lower compared to those without SPS after elective cardiac surgery. We observed a markedly increased mortality rate for patients with SPS from the first year of follow-up to 10 years postoperatively. Further, we demonstrated that SPS independently predicts a decreased survival rate, with a magnitude similar to well-known risk factors such as diabetes, COPD, renal dysfunction and decreased left ventricular function^26–29^.

In addition, our results seem to hold true regardless of mean eGFR. The HR for SPS as a predictor of mortality ranged from 1.8 to 2.7 in the different eGFR strata, suggesting that SPS is not a marker for decreased GFR and chronic kidney disease but is a condition of its own. Even in patients with normal eGFR, the HR for patients with SPS was markedly increased, making SPS a strong, independent predictor for mortality after elective cardiac surgery.

As a novel finding, we can show that SPS occurs significantly more frequently in diabetic patients. In our Cox model without diabetes, SPS had a HR of 2.09 (95% CI 1.65–2.65). When we added diabetes to the model, the HR for SPS became 1.76 (95% CI 1.32–2.35). More surprisingly, in patients with SPS, the survival for those with diabetes did not differ significantly from patients without diabetes: it was equally poor for both groups (Fig. 4). The pathologic mechanism of SPS and its relation to diabetic glomerulopathy is unclear, but it is possible that the thickening of the glomerular basement membrane in diabetes is related to the development of SPS^18^. This could explain why SPS occurs more frequently in diabetic patients and be a possible shared pathway for both SPS and diabetes for mortality.

Since 2015, when SPS was first described by our group, patients with SPS regardless of population have shown increased mortality and/or morbidity^12–16,30^. In the setting of the SENIORLAB study, Purde et al. demonstrated that otherwise healthy seniors with SPS had a significantly lower survival rate compared to those without^14^. In a mixed patient population with measured GFR and known cause of death, Åkesson et al. found that SPS was associated with higher mortality rates for cancer, cardiovascular disease, diabetes and chronic kidney disease with a HR of 3.2. Further, they demonstrated that patients with SPS had a markedly increased all-cause mortality when the measured GFR was normal^17^. SPS influence on mortality on patients undergoing CABG was investigated shortly after SPS was first described, and SPS was independently associated with decreased survival rates for the first time in a cardiac surgery population and the definition as well as the cut-off value for SPS was tried to be elucidated in that same population^12^. Ljungberg et al. found that patients suffering from SPS had a higher risk of future surgery for aortic stenosis^30^, and Christensson et al. demonstrated that in a heart failure population, the presence of SPS was associated with an increased risk of having right ventricle systolic dysfunction, even though eGFR did not differ between the groups^15^. Recently, den Bakker et al. found evidence of SPS occurring in children, although clinical data was not reported^21,31^. As these cases demonstrate, SPS is found in other patient groups besides cardiac surgery. Further, as SPS appears whenever a material is analyzed, it is reasonable to assume that SPS is far more widespread than has been documented.

Attempts to clarify the pathophysiology of SPS has recently been initiated. One pathophysiological model suggests that 5–30 kDa proteins, which include cystatin C, are retained and not excreted by the kidneys due to the shrinking of glomerular pores^19–21^. Evidence for this was presented by Sällman-Almén et al.^16^ who in studies of the proteome of SPS patients found raised levels of proteins with a mass of 5–30 kDa, and several of these proteins represented signaling molecules of atherosclerosis^16,20,21,32^. A recent manuscript (submitted but unpublished) studying diabetic patients undergoing kidney biopsies demonstrates that the thickening of the basal membrane in the glomerulus impedes the clearance of cystatin C but not creatinine^18^. However, the mechanisms behind the basal membrane thickening and glomerular pore shrinking is not yet fully understood.

When SPS was initially described, a cut-off ratio of 0.6 (eGFR_cystatin C_ ≤ 60% eGFR_creatinine_), derived from previous studies on pre-eclampsia in pregnant women, was somewhat arbitrarily used^19^. In a previous report, our group identified that the optimal cut-off for diagnosing SPS to maintain a high specificity of 95% was close to 0.6 with the CKD-EPI Eqs.^13^. In this report we treat SPS as a dichotomous variable, but similar results were found when we treated SPS and eGFR as continuous variables (data not presented).

Patients afflicted by SPS were significantly older, often had other classical risk factors and had longer cross-clamp and bypass times, all of which may serve as surrogate markers for intraoperative complexity. We could not detect a large difference between the sexes, as both male and female patients seem to be afflicted in largely the same ratios and with similar HR. The global burden of chronic kidney disease is substantially higher in women, further underscoring that SPS may have another pathophysiologic background than other types of chronic kidney disorders. The alternative cystatin C- and creatinine based GFR-estimating equations CAPA and LMRev showed similar results as when the CKD-EPI estimating equations were used.

The current retrospective study has both inherent strengths and weaknesses. Among the strengths are that it is a large, well-defined population from a single center yielding data with a high granularity. In addition, this population has both a high incidence of SPS and a high yearly risk for death, resulting in strong material from a statistical standpoint. Another advantage is the long and reliable follow-up. A patient population that is homogenous, however, also limits this study, as a larger and more heterogeneous population may yield different results.

In summary, we have demonstrated that SPS is a risk factor of equal importance to classical risk factors for long-term survival. To date, SPS has been found in healthy seniors, mixed patient populations and patients with cardiovascular disease. In all these groups, SPS has been shown to markedly decrease survival rates. Together with our finding that SPS is a strong predictor for premature death, SPS may well be a wide-spread and silent disease that could affect many millions.

## Supplementary Information

Supplementary Information.

## Data Availability

The datasets generated and/or analysed during the current study are not publicly available due the ethical approval does not allow sharing of data (LU EPN 2016/53) but are available from the corresponding author on reasonable request.
