# Supplementary material for: Reduced renal elimination of larger molecules is a strong predictor for mortality
Source: Sci Rep. 2022 Oct 20;12:17517. doi: 10.1038/s41598-022-22433-4 (PMC9584920; doi:10.1038/s41598-022-22433-4)
Supplement: Supplementary file 1 — Supplementary Information. [file 41598_2022_22433_MOESM1_ESM.docx]

**Supplementary material**

**SPS and diabetes in different strata for pre-operative renal function**

Of patients with mean eGFR <30 ml/min/1.73 m^2^, 13 (20%) of those with diabetes presented with SPS while 9 (11.25%) of non-diabetic patients presented with SPS (p=0.14). Of patients with mean eGFR 30-60 ml/min/1.73 m^2^, 63 (20%) of those with diabetes presented with SPS while 96 (13.62%) of non-diabetic patients presented with SPS (p=0.009). Of patients with mean eGFR >60 ml/min/1.73 m^2^, 45 (7.36%) of those with diabetes presented with SPS while 70 (3.16%) of non-diabetic patients presented with SPS (p<0.001).

**Alternative equations for estimating GFR**

When GFR estimating equations CAPA (cystatin C based) and LMrev (creatinine based) was employed with the same diagnostic cut-off ratio of eGFR CAPA ≤ 60% of eGFR LMrev, 92 patients were afflicted by SPS, yielding a prevalence of 2.3%. Overall, 1-, 5-, and 10-year survival was 88%, 46% and 38% respectively for patients with SPS and 97%, 87%, 78% for patients without SPS. The difference in survival was statistically significant (p<0.001 in 1-, 5- and 10-year survival). Our main Cox model was used which showed that the two risk factors with highest hazard ratios and significance were SPS (HR 1.66 (1.25-2.21) and mean eGFR CAPA LMrev <60 ml/min/1.73 m^2^ (HR 3.11 (2.70-3.58)).

**Supplementary Table 1 – Variables used in the analysis**

| **Variable** | **All**  **(3993)** | **SPS**  **(296)** | **No SPS (3697)** | **P-value (T-test/** χ**^2^ test)** |
| --- | --- | --- | --- | --- |
| Age (years) | 68 (±10) | 73 (±9.4) | 68 (±10) | 0.000 |
| Anemia | 915 (23%) | 131 (44%) | 784 (21%) | 0.000 |
| Female | 930 (23%) | 67 (23%) | 863 (23%) | 0.781 |
| Mean eGFR CKD-EPI | 69 (±20) | 54 (±16) | 71 (±19) | 0.000 |
| eGFR CKD-EPI_creatinine_ | 75 (±20) | 70 (±20) | 75 (±20) | 0.000 |
| eGFR CKD-EPI_cystatin C_ | 64 (±22) | 37 (±12) | 66 (±21) | 0.000 |
| Previous stroke | 375 (9.4%) | 50 (17%) | 325 (8.8%) | 0.000 |
| Diabetes | 990 (24.8%) | 121 (41%) | 869 (24%) | 0.000 |
| COPD | 449 (11.2%) | 55 (19%) | 394 (11%) | 0.000 |
| Previous vascular surgery | 146 (3.7%) | 26 (8.8%) | 120 (3.2%) | 0.000 |
| Leucocytes (x10^9^/L) | 8.0 (±3.6) | 8.5 (±2.9) | 8.0 (±3.6) | 0.021 |
| Unstable preoperative state | 131 (3.3%) | 12 (4.1%) | 119 (3.2%) | 0.452 |
| Peripheral vascular disease | 405 (10%) | 63 (21%) | 342 (9.3%) | 0.000 |
| LVEF <30% | 255 (6.4%) | 55 (19%) | 200 (5.4%) | 0.000 |
| Previous myocardial infarction | 1575 (39%) | 150 (51%) | 1425 (39%) | 0.000 |
| EUROSCORE I | 4.7 (±3.0) | 7.0 (±3.4) | 4.5 (±2.9) | 0.000 |
| Hemoglobin (g/L) | 135 (±16) | 128 (±20) | 136 (±16) | 0.000 |
| Thrombocytes (x10^9^/L) | 236 (±69) | 242 (±86) | 235 (±68) | 0.096 |
| CRP (mg/L) | 7,8 (±20) | 18 (±37) | 7 (±18) | 0.000 |
| IABP preoperative | 45 (1.1%) | 9 (3%) | 36 (1%) | 0.001 |
| **Peri-/post-procedural variables** | | | | |
| CABG | 3179 (80%) | 212 (72%) | 2967 (80%) | 0.000 |
| sAVR | 532 (13%) | 47 (16%) | 485 (13%) | 0.179 |
| CABG and sAVR | 282 (7.1%) | 37 (13%) | 245 (6.6%) | 0.000 |
| Heart-lung machine time (min) | 80 (±37) | 80 (±39) | 86 (±37) | 0.007 |
| Cross clamp (min) | 51 (±24) | 55 (±29) | 51 (±24) | 0.004 |
| Time on ventilator (h) | 10 | 9.7 | 16 | 0.001 |
| Atrial fibrillation | 889 (24%) | 77 (30%) | 812 (24%) | 0.027 |
| IABP postoperatively | 57 (1.4%) | 12 (4%) | 45 (1.2%) | 0.000 |
| Renal replacement therapy | 48 (1.3%) | 8 (2.9%) | 40 (1.2%) | 0.01 |
| On warfarin or NOAC | 893 (22%) | 85 (29%) | 808 (22%) | 0.006 |
| Aspirin | 3452 (86%) | 256 (86%) | 3196 (86%) | 0.985 |
| On clopidoprel or ticagrelor | 710 (18%) | 59 (20%) | 651 (18%) | 0.729 |
| Perioperative MI | 24 (0.6%) | 0 (0%) | 24 (0.7%) | 0.177 |
| Mediastinitis | 53 (1.4%) | 11 (4.2%) | 42 (1.2%) | 0.000 |
| Stroke | 12 (0.3%) | 0 (0%) | 12 (0.3%) | 0.341 |
| Reexploration for bleeding | 95 (2.4%) | 23 (7.8%) | 72 (1.9%) | 0.000 |

**Supplementary Table 1.** Variables used in the analysis presented whole as well as grouped on the presence of SPS. Described as mean (±SD) or number (%). P-levels from Student’s T-test or χ^2^-test depending on the data. CABG, coronary artery bypass surgery; COPD, chronic obstructive pulmonary disease; eGFR, estimated glomerular filtration rate and in ml/min/1.73 m^2^; IABP, intra-aortic balloon pump; LVEF, left ventricular ejection fraction; MI, myocardial infarction; NOAC, novel oral anticoagulants; sAVR, surgical aortic valve replacement; SPS, shrunken pore syndrome.

**Supplementary Table 2 – Cox gender specific multivariable analysis**

|  | **Female** | | **Male** | |
| --- | --- | --- | --- | --- |
|  | P-level | HR (95% CI) | P-level | HR (95% CI) |
|  |  |  |  |  |
| SPS | <0.001 | 1.94 (1.36-2.76) | <0.001 | 1.95 (1.58-2.43) |
| Diabetes | 0.431 | 1.11 (0.85-1.46) | 0.196 | 1.12 (0.94-1.33) |
| COPD | 0.125 | 1.28 (0.93-1.76) | 0.002 | 1.41 (1.13-1.75) |
| Anemia | 0.037 | 1.38 (1.02-1.86) | <0.001 | 1.68 (1.32-2.12) |
| Peripheral arterial disease | 0.010 | 1.50 (1.10-2.03) | <0.001 | 1.62 (1.32-2.00) |
| LVEF <30% | 0.016 | 1.68 (1.10-2.57) | <0.001 | 1.49 (1.17-1.89) |
| Leucocytosis | 0.384 | 1.12 (0.86-1.46) | 0.002 | 1.29 (1.10-1.53) |
| eGFR<60 ml/min/1.73 m^2^ | <0.001 | 2.46 (1.89-3.21) | <0.001 | 2.95 (2.50-3.47) |

**Supplementary Table 2.** Cox multivariable analysis of risk factors for mortality in females and males. COPD, chronic obstructive pulmonary disease; eGFR, estimated glomerular filtration rate and in ml/min/1.73 m^2^; LVEF, left ventricular ejection fraction; SPS, shrunken pore syndrome.

**Supplementary Table 3 – Cox diabetes specific multivariable analysis**

|  | **Diabetes** | | **Non-diabetic** | |
| --- | --- | --- | --- | --- |
|  | P-level | HR (95% CI) | P-level | HR (95% CI) |
|  |  |  |  |  |
| SPS | <0.001 | 1.76 (1.32-2.35) | <0.001 | 2.09 (1.65-2.65) |
| Female | 0.46 | 1.10 (0.85-1.44) | 0.12 | 1.16 (0.96-1.38) |
| COPD | 0.047 | 1.33 (1.00-1.78) | 0.003 | 1.41 (1.12-1.77) |
| Anemia | <0.001 | 1.66 (1.25-2.23) | 0.002 | 1.47 (1.15-1.88) |
| Peripheral arterial disease | 0.006 | 1.46 (1.11-1.91) | <0.001 | 1.67 (1.34-2.07) |
| LVEF <30% | <0.001 | 1.82 (1.33-2.49) | 0.004 | 1.35 (1.02-1.78) |
| Leucocytosis | 0.11 | 1.22 (0.96-1.54) | 0.012 | 1.25 (1.05-1.48) |
| eGFR<60 ml/min/1.73 m^2^ | <0.001 | 2.47 (1.92-3.17) | <0.001 | 3.00 (2.53-3.55) |
|  |  |  |  |  |

**Supplementary Table 3.** Cox multivariable analysis of risk factors for mortality in diabetic and non-diabetic patients. COPD, chronic obstructive pulmonary disease; eGFR, estimated glomerular filtration rate and in ml/min/1.73 m^2^; LVEF, left ventricular ejection fraction; SPS, shrunken pore syndrome.

**Supplementary Table 4 – Collinearity statistics**

|  | Collinearity statistics | |  |
| --- | --- | --- | --- |
|  | Tolerance | VIF | |
| Leucocytosis | 0.980 | 1.021 | |
| Diabetes | 0.958 | 1.043 | |
| COPD | 0.976 | 1.025 | |
| Anemia | 0.915 | 1.092 | |
| Peripheral arterial disease | 0.953 | 1.049 | |
| LVEF <30% | 0.964 | 1.038 | |
| eGFR<60 ml/min/1.73 m2 | 0.916 | 1.092 | |

**Supplementary Table 4.** Linear regression of collinearity statistics with SPS as dependent variable. COPD, chronic obstructive pulmonary disease; eGFR, estimated glomerular filtration rate and in ml/min/1.73 m^2^; LVEF, left ventricular ejection fraction; SPS, shrunken pore syndrome; VIF, variable inflation factors.

**Supplementary Figure 1 – Survival for females after cardiac surgery based on SPS**


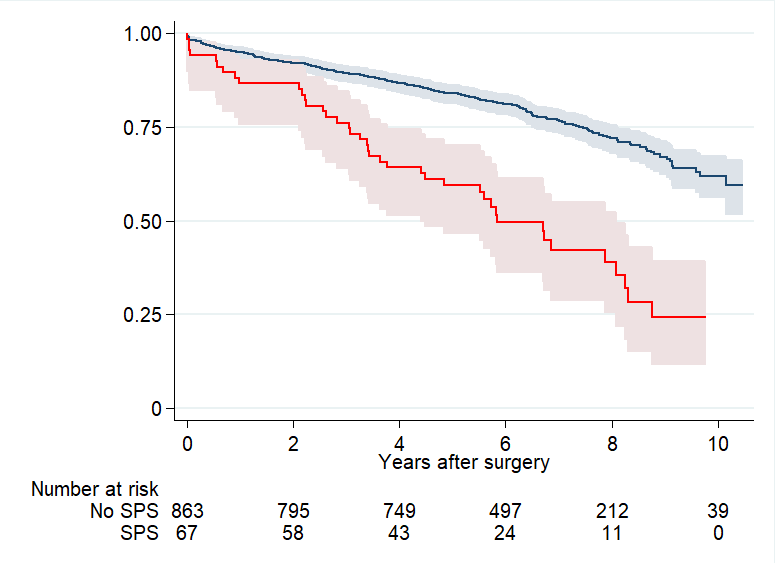


**Supplementary Figure 1.** Survival after elective cardiac surgery for female patients with Shrunken Pore Syndrome (SPS, red solid line) and without (blue solid line). Faded area represents 95% CI.

**Supplementary Figure 2 – Survival for males after cardiac surgery based on SPS**


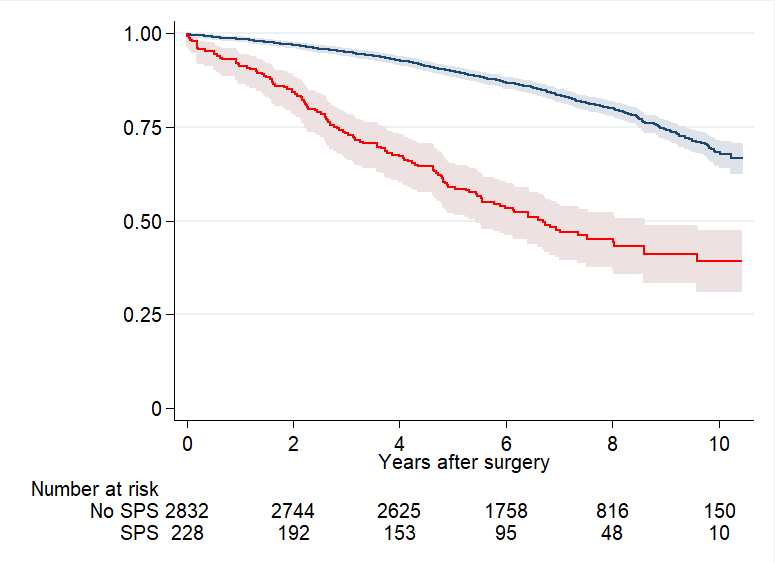


**Supplementary Figure 2.** Survival after elective cardiac surgery for male patients with Shrunken Pore Syndrome (SPS, red solid line) and without (blue solid line). Faded area represents 95% CI.
